# Anthelmintics Resistance; How to Overcome it?

**Published:** 2013

**Authors:** Hatem A. Shalaby

**Affiliations:** Department of Parasitology and Animal Diseases, National Research Center, Giza, Egypt

**Keywords:** Anthelmintic, Resistance, Helminths, Control

## Abstract

Many parasitic helminthes of veterinary importance have genetic features that favor development of anthelmintic resistance, this becoming a major worldwide constrain in livestock production. The development of anthelmintic resistance poses a large threat to future production and welfare of grazing animals. Development of variable degrees of resistance among different species of gastrointestinal nematodes has been reported for all the major groups of anthelmintic drugs. It has been observed that frequent usage of the same group of anthelmintic; use of anthelmintics in sub-optimal doses, prophylactic mass treatment of domestic animals and frequent and continuous use of a single drug have contributed to the widespread development of anthelmintic resistance in helminthes. The degree and extent of this problem especially with respect to multidrug resistance in nematode populations is likely to increase. Maintaining parasites in refugia and not exposed to anthelmintics, seems to be a key point in controlling and delaying the development of resistance, because the susceptible genes are preserved. Targeted selective treatments attract the interest of scientists towards this direction. Additionally, adoption of strict quarantine measures and a combination drug strategy are two important methods of preventing of anthelmintic resistance. Experience from the development of anthelmintic resistance suggests that modern control schemes should not rely on sole use of anthelmintics, but employ other, more complex and sustainable recipes, including parasite resistant breeds, nutrition, pasture management, nematode-trapping fungi, antiparasitic vaccines and botanical dewormers. Most of them reduce reliance on the use of chemicals and are environmental friendly. Finally, if new anthelmintic products are released, an important question will be raised about how they should be used. It is suggested that slowing the development of resistance to a new class are likely to be gained by releasing it in combination with one or more of the older anthelmintic classes, especially where efficacy of the older active(s) remains high.

## Introduction

Helminths are a diverse group of parasitic worms, encompassing nematodes, cestodes and trematodes, and constitute a major health problem for humans and animals in many parts of the world (1, 2). Although their diseases impact could be reduced dramatically by improved sanitation for humans and pasture control in domestic animals, such methods are not sufficient to eradicate these parasites. In the absence of vaccines, control of these parasites is reliant on chemotherapy to ease symptoms and reduce transmission. The intensive use of drugs in the live-stock industry has led to widespread resistance to all current anthelmintics (3). With few new drugs or vaccines, the fight against parasites could become a losing battle. Understanding the development of drug resistance in parasitic helminths is crucial to prolonging the efficacy of current anthelmintics and developing markers for monitoring drug resistance. It will also be beneficial in the design of new chemotherapeutic agents to overcome or prevent resistance and the identification of new drug targets (4). In order to be able to provide solutions for the threats provoked by the progressing spread of anthelmintic resistance (AR), mainly in livestock, a number of urgent questions remain to be answered. These questions focus on the factors contributing towards AR, methods for the detection of resistance and some possible solutions to control the development of AR. Despite notable ongoing activities to identify and evolve new anthelmintic classes by a shrinking list of institutions, there is doubt that we will see the release of a product with a new mode of action in the livestock area in the near future (1). But even if new drugs are developed, these will inevitably also be affected by the problem of AR in due course. Therefore, it is of ultimate importance to find better ways to use the anthelmintic substances we do have or will have in a most sustainable manner, preserving their efficacy as long and in as many parasite species as possible.

As it would go beyond the scope of this review, a list of important aspects with potential to contribute to solve the problem of AR could be addressed.

### What is anthelmintic resistance (AR)?

Helminthic diseases are treated with a variety of drugs including macrocyclic lactones, benzimidazoles, imidazothiazoles and praziquantel. In animals, resistance to anthelmintics occurred rapidly after their introduction. There is considerable debate about the definition of resistance, and ‘tolerance’ is used to describe the stage between success and failure of drug treatment. However, as stated by Coles (5), resistance occurs when a susceptible population shows any decrease in response to treatment and is complete when the maximum dose of drug that can be tolerated by the host has no effect. Unfortunately, the reduction in response can manifest in different ways, either as a heritable decline in the efficacy of an anthelmintic against a population of parasites that is generally susceptible to that drug or as a decrease in the time a drug treatment exerts its effect, with resistant populations requiring more frequent treatments than previously administered. In an attempt to provide a scientific basis for resistance, resistance has been identified by an increase in the proportion of organisms in a population carrying a gene demonstrated to be linked with resistance (6). These heritable changes can be either genetic (including mutations, deletions or amplifications of specific genes) or epigenetic (where by methylation of genes or promoter regions of the genes change the gene expression in response to the drug) (7).

### The extent of the problem

In small ruminants, anthelmintic-resistant nematodes are already a serious problem (8). In Australia, for example, the prevalence and severity of resistance threatens the profitability of the entire sheep industry (9). Resistance has arisen to all of the major families of broad spectrum anthelmintics (10), the benzimidazoles (BZ), levamisole (LEV) and the other nicotinic agonists, in addition to the avermectins and milbemycins (AM) (including ivermectin, doramectin and moxidectin). Nematodes that are resistant to other, narrow-spectrum anthelmintics, such as closantel, have also been reported (10). The situation in cattle is currently less severe, but there are cattle nematodes resistant to multiple anthelmintic classes in New Zealand and South America (11, 12) and this will probably become more widespread. In horses, BZ resistance is that which widespread among the cyathostomins. The AM are still effective for cyathostomins, but not for *Parascaris* in foals (13, 14). This could change as AM are used more frequently and selection pressure increases.

Although resistance in flukes has not yet reached the levels present in nematodes, resistance exists for the salicylanilides, rafoxanide and closantel, with evidence of cross-resistance to the halogenated phenol, nitroxynil (15). Of greater concern is the spread of resistance to triclabendazole, the main drug used to treat fluke infections because of its high activity against the migrating immature stages. Resistance was first reported in Australia in 1995 (16) and has since been described in The Netherlands, UK and Ireland. At the same time, there has been a dramatic resurgence of fasciolosis as a result of climate change and the advent of milder, wetter weather (17).

Anthelmintic resistance is a threat to agricultural incomes, and has been reported from all the four corners of the world, to all available drugs, in all classes of helminthes (18).

### Development of anthelmintic resistance

The general consensus is that anthelmintic resistance appears to be a pre-adaptive heritable phenomenon with the gene or genes conferring resistance being present within the parasite population even prior to the drug being used for the first time (19). Under these circumstances resistance arises as a result of selection through exposure of the worm population to an anthelmintic. When an animal is optimally exposed to an anthelmintic the only worms that should survive are those that carry the genes that confer resistance. For a short period (until the animal becomes re-infected with drug susceptible worms from pasture) the resistant survivors are the only worms laying eggs and in this way the gene pool for resistance is increased. The rate of development of resistance is influenced by many factors, of them, significant ones are described here.

### Treatment frequency

It has been observed that frequent usage of the same group of anthelmintic may result in the development of AR (20). There is evidence that resistance develops more rapidly in regions where animals are dewormed regularly. Anthelmintic resistance in *H*. *contortus* has been reported in some humid tropical areas where 10 to 15 treatments per year were used to control this parasite in small ruminants (21). Drug resistance, however, can also be selected at lower treatment frequencies, especially when the same drug is used over many years. Coles (22) have reported the development of AR even when only two or three treatments were given annually.

### Anthelmintics underdosing

Underdosing is generally considered an important factor in the development of AR because subtherapeutic doses might allow the survival of heterozygous resistant worms (23). Several laboratory experiments have shown that underdosing contributes to the selection of resistant or tolerant strains (24). Moreover, variation in bioavailability in different host species also is crucial for making a decision about correct dose. Some indirect field evidence further supports this conclusion. For an example, the bioavailability of benzimidazole and levamisole is much lower in goats than in sheep, resultantly those goats should be treated with dosages 1.5 to 2 times higher (the single dose is much less inferior than “sub-optimal”, it is rather near half the dose necessary for goats) than those given to sheep (25). For many years, however, sheep and goats were given the same anthelmintic doses. The fact that AR is very frequent and widespread in goats may be a direct consequence of difference in metabolism of drugs. To reduce the costs of anthelmintic treatment in developing countries, the use of lower dosages than the recommended therapeutic ones has been advocated. Such practices should clearly be avoided. Most of the currently applied anthelmintics are in fact subcurative in at least part of the population. Additionally, there are a number of species of nematodes which are present as mixed infection in animals throughout the world which respond to different groups of anthelmintics differently due to the irregular susceptibility of these species to a given anthelmintic. This is considered acceptable for morbidity control, but in the long run such strategies may contribute to the development of AR as well (26).

### Mass treatment

Prophylactic mass treatments of domestic animals have contributed to the widespread development of AR in helminths. Computer models indicate that the development of resistance is delayed when 20% of the flock is left untreated (27) but it needs confirmation through experimentation. This approach would ensure that the progeny of the worms surviving treatment will not consist only of resistant worms. Leaving a part of the group untreated; especially the members carrying the lowest worm burdens should not necessarily reduce the overall impact of the treatment. In worm control in livestock, regular moving of the flocks to clean pastures after mass treatment and/or planning to administer treatment in the dry seasons is a common practice to reduce rapid reinfection. However, these actions result in the next helminth generation that consists almost completely of worms that survived therapy and, therefore, might contribute to the development of AR (20, 23).

### Single-drug regimens

Frequent and continuous use of a single drug leads to the development of resistance. For example, a single drug, which is usually very effective in the first years, is continuously used until it no longer works (28). In a survey of sheep farmers in Tennessee, (29) found that one out of every two flocks was dosed with a single anthelmintic until it failed. Long-term use of levamisole in cattle also led to the development of resistance, although the annual treatment frequency was low and cattle helminthes seemed to develop resistance less easily than do worms in small ruminants (30). Frequent use of ivermectin without alternation with other drugs has also been reported as the reason for the fast development of resistance in *H*. *contortus* in South Africa and New Zealand (31, 32).

### Transmission of resistance

Studies examining changes in the prevalence of anthelmintic resistance have suggested that initially “on farm” selection is the crucial process. However, as resistant parasite populations become more common, animal movement is one of the key factors that account for the rapid changes that occur during the last stages of the development process. There have been several well-documented examples of international transmission of resistance in sheep and goats (33).

### Prevention of anthelmintic resistance

The problem of AR can be circumvented either by delaying its onset or use of alternate strategies in the form of integrated parasite management.

### Delaying the onset of anthelmintic resistance Refugia

From a clinical standpoint, it is important to appreciate that resistance is a genetic trait that only becomes expressed phenotypically once allele frequencies of resistance genes reach fairly high levels. Benzimidazole resistance could not be detected using phenotypic-based assays (e.g., egg hatch or fecal egg count reduction tests) until 25% of the gastrointestinal nematodes were resistant (34). Therefore, prevention of resistance must be aimed at reducing the rate with which resistance alleles accumulate, and strategies designed to slow the development of resistance must be in integrated early on in the process of resistance evolution, before there is any clinical evidence of reduced drug effect. This is accomplished best by following practices that ensure maintenance of an adequate level of refugia; a term used to describe the proportion of a parasite population that is not exposed to a particular drug, thereby escaping selection for resistance.

Most parasitologists now consider levels of refugia as the single most important factor contributing to selection for anthelmintic resistant parasites (27). Worms in refugia provide a pool of genes susceptible to anthelmintics, thus diluting the frequency of resistant genes. As the relative size of the refugia increases, the rate of evolution toward resistance decreases. In gastrointestinal nematodes of small ruminants, which have a direct life cycle, refugia are supplied by: 1) stages of parasites in the host that are not affected by the drug treatment, 2) parasites residing in animals that are left untreated with a particular drug, and 3) free-living stages in the environment at the time of treatment. For many years, parasitologists and veterinarians have recommended that all animals should be treated with an anthelmintic at the same time. However, this strategy has turned out to be unsustainable, and parasitologists now favor a selective approach where only animals in need of treatment actually receive medication. This selective approach is highly compatible with host parasite dynamics; parasite burdens are highly aggregated in hosts, with 20–30% of animals harboring 80% of the worms (35). Treatment of animals with low worm burdens does little to control parasites, but removes an important source of refugia, thereby accelerating the evolution of resistance. Climatic conditions have fundamental effects on the numbers in refugia. Few free-living stages survive in arid climates, so the pasture refugium is small. The appearance of avermectin resistance in *Telodorsagia* spp. in Western Australia after only two treatments with the drug illustrates the power of selection in arid areas (36). Cattle dung pats can also represent a reservoir of infective larvae for up to 12 months, ensuring a large refugium and slow selection for resistance in cattle parasites.

### Adoption of strict quarantine measures

Effective management strategies to prevent development of anthelmintic resistance are worthless if producers purchase resistant worms residing in breeding stock. Therefore, strict quarantine procedures should be instituted for all new additions. This practice is more important than ever, as in recent years several farms with high-quality breeding stock dispersed herds where *H*. *contortus* and *T*. *colubriformis* were resistant to benzimidazoles and moxidectin. There is no faster way to spread resistance than to bring gastrointestinal nematodes to a farm. The current recommendation is to quarantine (on dry lot where feces can be removed) every new addition, dose with triple-class anthelmintic therapy, and perform fecal egg count reduction tests. Feed should be withheld for 24 hours before treatment, then moxidectin, levamisole, and albendazole should be administered consecutively (do not mix drugs together) at the appropriate dose for sheep or goats. Fourteen days later, treated animals should be evaluated by fecal egg count and fecal flotation techniques. The fecal egg count should be zero, and flotation should yield very few or no eggs. Furthermore, after receiving this treatment, animals should be placed on a contaminated pasture. Never should an animal be placed onto a clean pasture after a triple anthelmintic class treatment regimen is administered, because any surviving worms will be triple resistant and there will be no refugia on pasture to dilute the future transmission of any eggs that are shed (37).

### A combination drug strategy

Treating simultaneously with 2 drugs from different anthelmintic classes is one method of preventing the development of anthelmintic resistance. A computer based model has documented that if this strategy is used when the drugs are first introduced, before there is any selection for resistance to either drug, appreciable resistance will not develop for over 20 years. However, once resistance alleles accumulate in worm populations, this strategy will probably not be successful. Compared with individual drug effects, anthelmintics of different chemical classes administered together induce a synergistic effect, resulting in clinically relevant increases in the efficacy of treatment. This synergistic effect is most pronounced when the level of resistance is low. Once high-level resistance to both drugs is present, the synergistic effect is unlikely to produce acceptable levels of efficacy. In contrast, the same model indicated that rotating drugs with each treatment, using annual rotation or a 5- or 10-year rotation resulted in high-level resistance within 15 to 20 years (38). Thus, the common recommendation of annual rotation must be challenged. Rotation of drugs was originally suggested on the basis of the hypothesis that reversion to susceptibility (or at least substantial decrease in resistance gene allele frequency) might occur if resistant worms were less fit than were susceptible worms, and counter selection was applied via treatment with a drug from a distinct chemical class. However, evidence that resistant worms are any less fit or that true reversion occurs in the field is scant. Despite this, the concept of rotation is often viewed as a bona fide resistance prevention scheme, which it is not. Therefore, some leading small ruminant parasitologists are now calling for an end to the practice of rotation (27). It is suggested that a drug should be used until it is no longer effective, then a different drug should be used. The main rationale behind this recommendation is that: 1) the arsenal of effective drugs is limited, making it difficult to institute a true rotation on many farms; and 2) progressive development of resistance will make it easier to monitor the resistance problem on a farm.

Synergistic combinations have been described for both human and veterinary infections. For example, combinations of praziquantel with oxamniquine or artemether have been shown to be synergistic for the treatment of schistosome infections (39, 40). Synergism between albendazole and ivermectin or diethylcarbamazine, and between mebendazole and levamisole or pyrantel has been described for the treatment of soil-transmitted helminths (40). For veterinary parasites, a combination of mebendazole and levamisole has been shown to be synergistic against *H. contortus* in sheep (41), febantel and pyrantel against *Ancylostoma caninum* in dogs (42), *Heterakis spumosa* (43) in mice, and febendazole and pyrantel against *Toxocaracanis in vitro* (44). For the nematodes of small ruminants, the use of combinations serves dual purposes (45): 1) to maintain nematode control in the presence of AR, sometimes involving more than one parasite species and/or more than one class of anthelmintic; and concurrently, 2) to delay the development of AR to the component chemical classes in those species in which resistance is not yet evident. Without the use of combinations, some anthelmintic classes could no longer be used on many farms, despite still being highly effective against a large proportion of the parasite species on these farms. Studies to date have shown that the use of combinations provides more sustainable control of sheep nematodes than using them separately, either sequentially or in different patterns of rotation, and this occurred, albeit to a lesser extent, even in the presence of cross-resistance between the two anthelmintic classes or moderate levels of pre-existing resistance to one of the classes (46). For the liver fluke, resistance to TCBZ was first identified in Australia and appears to be spreading throughout Europe (47). A combination of TCBZ with either clorsulon or luxabendazole has been shown to be effective against Six-week-old triclabendazole-resistant flukes. Other combinations of drugs are active against salicylanilide-resistant *F*. *hepatica* (48) who mentioned that the efficacy of closantel was enhanced by combining the drug with either clorsulon or luxabendazole when the drugs were used at a fraction of their respective recommended dose rates. The combination of a slightly increased dose rate of closantel with a low dose rate of clorsulon showed strong synergistic effect and achieved high efficacy against a salicylanilide resistant strain of *F. hepatica* aged 4 weeks. With some adjustments of dose rates, combination products can be developed which are highly effective against *Fasciola* spp. aged 2 weeks and older. In certain combinations, one or both of the active components have additional effect against parasitic infections other than fasciolosis. Some combinations would be suitable for the treatment of resistant and susceptible strains of trematodes (*Fasciola* spp., *Dicrocoelium* spp. and *Eurytrema pancreaticum*) as well as gastrointestinal nematodes, lungworms, tapeworms and *Oestrus ovis* in sheep. Salicylanilides act on both *F. hepatica* and *H. contortus* by uncoupling oxidative phosphorylation and related reactions of the mitochondrial membranes involved in electron transport. In the development of resistance a permeability barrier may operate (49). The regular use of these drugs may play an important role in the development of salicylanilide resistance for both parasites. A level of resistance of *H. contortus* to rafoxanide and closantel has been reported with references to previous investigations by Rolfe et al. (50). Since the mode of action of salicylanilides is similar in either *Fasciola* spp. or *H contortus*, the closantel–luxabendazole combination would be effective against the salicylanilide resistant strains of the two parasites, with an additional broad-spectrum activity against gastrointestinal nematodes. All combinations with closantel would give persistent efficacy against susceptible *H. contortus*. Recently, Shalaby et al. (51) carried out an in vitro study to investigate the comparative morphological effects of ivermectin/*Nigella sativa* oil combination and each of them on its own against helminth parasites; *H. contortus* (nematode), *Moniezia expansa* (cestode) and *F*. *gigantica* (trematode). This study had provided morphological evidence for the greater anthelmintic activity of ivermectin on combination with *N. sativa* oil, and the results lent support to the idea of using drug combinations against helminthes infections.

### Alternate strategies Genetic improvement

There is considerable evidence that part of the variation in resistance to helminths infection is under genetic control. Resistance is most likely based on inheritance of genes that play a principal role in expression of host immunity. Several breeds of sheep around the globe are known to be relatively resistant to infection. Using such breeds exclusively or in crossbreeding programs would certainly lead to improved resistance to worm infection, but some level of production might be sacrificed (52). Although such a strategy may be acceptable to some, selection for resistant animals within a breed also is a viable option. Within a breed, animals become more resistant to infection with age as their immune system becomes more competent to combat infection. Some animals within such a population do not respond well and remain susceptible to disease; therefore, the majority of the worm population resides in a minority of the animal population. It would make sense to encourage culling practices where these minority ‘‘parasitized’’ animals were eliminated, thus retaining more-resistant stock. This approach has been used successfully in some areas of New Zealand and Australia, but it may take a long time (up to 8–10 years) to achieve satisfactory results (53).

### Nutrition

The strongest link between nutrition and parasitism has been illustrated between protein intake and resistance to gastrointestinal nematode infection (54). The most dramatic has been abolishment of the periparturient egg increase in lambing ewes by providing protein at 130% of requirements. Immunity is closely related to protein repletion. Gastrointestinal nematodes increase the demand for amino acids by the sheep. Compared with uninfected lambs, those infected with gastrointestinal nematodes will voluntarily select a higher protein diet. There is conflicting documentation that sheep will decrease feed intake when initially infected with gastrointestinal nematodes. Some authors hypothesize that the decrease in intake may be attributable to stimulation of the immune system or that the host is becoming selective in its diet. Supplementation with phosphorus has been shown to prevent worm establishment. Cobalt deficiency also has been associated with reduced immunity to gastrointestinal nematodes. Adequate copper values are necessary for development of immunity to gastrointestinal nematodes. A promising work suggested that treatment of lambs with copper oxide wires orally reduced *H*. *contortus* burdens. However, copper toxicosis would be a concern associated with this treatment. Surprisingly, the addition of molybdenum at a concentration of 6–10 mg/d decreased worm burdens in lambs (55).

### Pasture management

Reducing exposure of susceptible hosts in control programs is paramount. The goal of pasture management is to provide safe pastures for grazing. A safe pasture is one that has not had sheep or goats grazed on it for 6 months during cool/cold weather or 3 months during hot, dry weather. Weaning sheep and goats at 2 months of age and rotating them through pastures ahead of the adults will minimize the exposure of susceptible animals to large numbers of infective larvae. Pastures should be subdivided into smaller lots to allow longer periods before regrazing. Pastures that have become heavily contaminated because of mismanagement can be tilled and reseeded. Stocking rate is an important consideration in parasite control as it affects exposure to infective larvae and contamination of the pasture. It is impossible to make a general recommendation on stocking rate as this will vary according to type of pasture, time of the year, current weather conditions, and type of animal being grazed. Thumb rules include 5–7 goats or 5 sheep being the equivalent of 1 cow, and suggestions of 5–7 goats/acre. Goats prefer to browse brush and trees, whereas sheep prefer to graze near the ground. Pasture management must include monitoring the condition of the herbage to ensure that overgrazing does not occur and to maintain a productive pasture (56).

In the early spring or at the onset to the rainy season, reduced pasture contamination is the most important aspect of control. Strategic deworming to remove arrested or recently emerged larvae before they contaminate the pasture will reduce pasture contamination. Treatment 2 weeks after a rain that removes recently acquired worms before they can begin passing eggs also will decrease pasture contamination. When plants high in condensed tannins are grazed, there is evidence that the incoming larvae are adversely affected as well as providing bypass protein for the host. If animals are allowed to browse, their chances of acquiring larvae diminishes as the distance from the ground increases. Most infective larvae are found within 2 inches (50 mm) of the soil surface (57, 58).

### Nematode-trapping Fungi (as a biological control agent)

The philosophy behind biological control is that by using one of the natural enemies of nematodes, it will be possible to reduce the infection level on pasture to a level at which the grazing animals avoid both clinical and subclinical effects due to parasitic nematodes. Although no biological control agent will eliminate the number of infective stages to zero, the grazing animals, such as sheep, will constantly receive a small amount of parasitic larvae and thereby should be able to develop a natural immune response. Research with nematode-trapping fungi has documented the potential as a biological control agent against the free-living stages under experimental and natural conditions (59). These fungi occur in the soil throughout the world where they feed on a variety of free-living soil nematodes. These fungi capture nematodes by producing sticky, sophisticated traps on their growing hyphae. Of the various fungi tested, *Duddingtonia* *flagrans*, has the greatest potential for survival in the gastrointestinal tract of ruminants. After passing through the gastrointestinal tract, spores germinate and looped hyphae trap the developing larval stages in the fecal environment. This technology has been applied successfully under field conditions in all livestock species, and is an environmentally safe biological approach for control of worms under sustainable, forage-based feeding systems (60).

The only delivery system is incorporating the fungal spores into supplemental feedstuffs that must be fed daily. This requires a management system that can accommodate daily feeding to ensure that all animals consume an equivalent amount of feed. To achieve adequate control of larvae in the feces during the transmission season, spores must be fed for a period of no fewer than 60 days. This can be expensive and time consuming. A bolus prototype is being developed that would allow a single administration where spores would then be slowly released over a 60- day period (59). Research has been conducted throughout the world covering many different climates and management systems. An Australian parasite model showed that if the fungus performs efficiently (≥90% reduction in worm burden) for 2 or 3 months, it should contribute significantly to a reduction in the number of dead lambs otherwise occurring when managed only by anthelmintic treatment and grazing management. Feeding or field trials have clearly demonstrated that dosing with a few hundred thousand spores per kilogram of live body weight (BW) not only reduced the number of infective larvae but also increased the BW of the lambs compared with controls not given fungus. In tropical Malaysia, small paddock trials and field studies resulted in significant improvements, in terms of lower worm burdens and increased live BW, when feeding half a million spores daily to grazing lambs. Additional benefits have been observed when the fungus is employed in combination with a fast rotational grazing system. Research has also demonstrated that spores can be delivered in slightly moist feed block material, but only if such blocks are consumed rapidly, because of their very short shelf life. In the northern, temperate Danish climate it has been demonstrated that daily feeding of half a million spores per kilogram of live BW can lead to significant production benefits, with increased live BW gain in fungus-exposed animals. Biological control of parasitic nematodes in sheep seems to hold promise for the future, but to be able to assist producers, the optimal delivery system needs to be refined and further developed (61).

### Antiparasitic vaccines

As a consequence of drug resistance, efforts have increased in recent years to develop functional vaccines. This has been made possible by newer technologies in gene discovery and antigen identification, characterization, and production. At present, only one worm vaccine is on the market for the cattle lung nematode *Dictyocaulus viviparous* (Bovilis Lung worm), consisting of irradiated infective L3 larvae that cannot develop into the adult stage (62). Vaccination with irradiated L3 larvae of the economically important gastrointestinal nematodes has been attempted but was not successful due mainly to their lack of efficacy in inducing immunity in young animals (63). The increasing drug resistance of gastrointestinal nematodes has renewed intense interest in developing vaccines for these important veterinary pathogens. The most promising vaccine for small ruminant worms is based on a ‘‘hidden gut’’ antigen and specifically targets *H*. *contortus* (64). This antigen is derived from the gut of the worm and, when administered to the animal, antibodies are produced. When the worm ingests blood during feeding, it also ingests these antibodies. The antibodies then attack the target gut cells of the worm and disrupt the worm's ability to process the nutrients necessary to maintain proper growth and maintenance, thus killing the worms. This vaccine has been tested successfully only in sheep under experimental conditions and has had limited success under field conditions. Reasons for this lack of success are unclear. The drawback to this vaccine is that the antigen is normally ‘‘hidden’’ from the host, and a number of vaccinations may be required to maintain sufficiently high antibody titer to combat infection. This process may be quite expensive. In addition, massive numbers of whole worms are necessary to extract limited amounts of antigen; therefore, this will only be practical when the antigen can be mass produced artificially via recombinant technology to lower costs. Vaccines for other worms that do not feed on blood have focused on using antigens found in worm secretory and excretory products. These antigens have contact with the host and should stimulate continuous antibody production. However, protection has been quite variable and marketing of such products has not been pursued.

The most important veterinary trematode species are liver flukes (*Fasciola hepatica* and *Fasciola gigantica*). Acquired resistance to a secondary *F. gigantica* infection following a primary infection or vaccination has been demonstrated in cattle, goats and sheep (65). In cattle, using irradiated metacercariae as the immunizing vaccine, Bitakaramire (66) reported a 98% reduction in worm burdens in vaccinated calves. Younis et al. (67), using a range of immunizing regimes, showed that vaccination of zebu calves with irradiated metacercariae reduced worm burdens by 45–68%. In goats, vaccination with a primary exposure to irradiated metacercariae reduces fluke burdens by 43% (68) and 82–85% (69). It is well established that sheep do not acquire resistance to *F. hepatica* as determined from the observed yields of mature parasites after primary and secondary infections with *F. hepatica*. In European sheep, yields of *F. hepatica* ranged from 16 to 38% after primary infection, and from 13 to 31% after secondary infection, indicating that resistance to *F. hepatica* does not develop in these sheep breeds (70). In contrast, acquired resistance to *F. gigantica* has been observed in sheep. A'Gadir et al. (71) reported a significant reduction in parasite numbers in Sudanese desert sheep vaccinated with irradiated metacercariae of *F. gigantica* where the recovery of adult parasites was reduced from 17% in control animals to 3.4% in the vaccinates.

There have been many attempts to vaccinate animals with various liver fluke extracts, such as crude somatic antigens, excretory/secretory antigens and various defined antigens. The mean level of reduction in worm burdens observed in cattle with different antigens was in the range of 43–72%, suggesting that the control of fasciolosis by immunological intervention may be an achievable goal (72). The search for the development of an effective vaccine against *Fasciola* has focused on essential enzymes. One of the most promising candidates has been glutathione S-transferase (GST) (73). The GST belongs to a family of enzymes that are involved in the cellular detoxification process. It primarily functions by catalyzing the conjugation of the glutathione to a wide variety of electrophilic toxic substrates (74). GSTs of helminths act as immune defense proteins and have significant activity with lipid peroxidation-derived carbonyls and also have the potential to neutralize exogenously derived toxins such as anthelminthics (75). GSTs have been highly conserved throughout evolution and are particularly abundant in parasitic helminthes. Whereas the homologous GST fraction purified from *F. hepatica* proved ineffective in a vaccination study in rats (76), a trial in sheep indicated that a mean 57% reduction in worm burdens was possible (77). However, a preliminary trial in cattle, using native GST from adult fluke emulsified in Freund's complete adjuvant, was not successful in inducing protection against fluke challenge and the lack of protection in this experiment was attributed to the production of considerably lower titers of anti-GST antibodies than in the sheep study (73). Indeed, significant reduction in fluke burdens (49–69%) was observed in cattle vaccinated with GST in Quil A/Squalene Montanide (73). Vaccine trials were conducted in goats by Degheidy et al. (78) evaluating the efficacy of three antigens of adult *F. gigantica*, as vaccines against fascioliasis. The antigens tested were crude worm, excretory-secretory material and GST and were emulsified in Freund's adjuvant. The results indicated that the highest reduction in eggs per gram feces (EPG) and fluke burden was observed in goats immunized with purified GST antigen (90.7 and 66.1%, respectively). Besides, this purified antigen induced the highest effect in minimizing fluke size among the tested antigens. This protection level in goats supported the notion of variable effect of vaccination with trematode GST in various ruminant species.

The mode of action of the immune response against GST which leads to parasite elimination remains to be determined. There appear to be at least two possibilities: (i) an antibody response directed to the active or ligandin site of GST neutralizes or reduces GST activity in the parasite by steric hindrance at substrate binding sites: this results in tissue damage in the fluke resulting from the exogenous action of reactive oxygen/nitric oxides released by the host inflammatory response on to the parasite; (ii) GST is acting as an abundant antigen released by the fluke which induces an inflammatory immune response which kills the parasite (73).

### Botanical dewormers

In last two decades, there has been a resurgence of interest in traditional health-care practices all over the world. These traditional practices involve diagnostics, herd grazing and pasture management as well as manipulation and treatment. The incidence of AR has simply forced veterinarians/producers to adopt alternative control strategies. Plants have been used from ancient times to cure diseases of man and animals. This system of therapy is commonly referred as ‘unani, folk, eastern, or indigenous’ medicine. The plant kingdom is known to provide a rich source of botanical anthelmintics, antibacterials and insecticides (79). A number of medicinal plants have been used to treat parasitic infections in man and animals. There are many plants which have been reported in the literature for their anthelmintic importance. Among the most common medicinal plants which have anthelmintic effect are *Allium sativum, Nigella sativa, Artemisia* spp., *Balanites aegyptiaca*, *Acacia* spp., cucurbile (pumpkin seeds), *Commiphora molmol* (Myrrh), *Calendula micrantha officinalis, Peganum harmala* and Tumeric (curcumina) (80–84).

Additionally, various pasture tanniferous plants have also been investigated for potential effect against either incoming parasite larvae and/or already established worms (85). It has been postulated that the beneficial effects of tanniferous plants against internal parasites could be due to one, or a combination, of the following factors:

- Tanniferous plants increase the supply and absorption of digestible protein by animals. This is achieved by tannins forming non-biodegradable complexes with protein in the rumen, which dissociate at low pH in the abomasums to release more protein for metabolism in the small intestine of ruminants – in other words, ”natures protected protein.” This indirectly improves host resistance and resilience to nematode parasite infections.
- Tannins have a direct anthelmintic effect on resident worm populations in animals.
- Tannins and/or metabolites in dung have a direct effect on the viability of the free-living stages (development of eggs to infective larval stages).

These plants can be a promising future for the control of worms which had previously shown resistance to synthetic drugs.

## Conclusion

AR is a threatening problem to livestock industry posing very threats to the future welfare and production of livestock throughout the world. The factors considered most significant have been an excessive frequency of treatments and the administration of an inadequate dose (underdosing) particularly latter is true for developing countries. It may be concluded that sustainable control strategies for helminthosis may require an integrated approach incorporating environmental management, and require a combination drug strategy in order to minimize the pressure for parasite adaptation.
